# High visibility colored fabrics for normal trichromats and individuals with color vision defects in a sunset-simulated environment

**DOI:** 10.1371/journal.pone.0274824

**Published:** 2022-09-16

**Authors:** Tatsuya Iizuka, Takushi Kawamorita, Hajime Tsuji, Hiroyuki Kanai, Toshihiro Hirai, Hiroo Suzuki, Tomoya Handa, Hitoshi Ishikawa

**Affiliations:** 1 Department of Vision Science, Kitasato University Graduate School of Medical Sciences, Kanagawa, Japan; 2 Department of Orthoptics and Visual Science, Kitasato University School of Allied Health Sciences, Kanagawa, Japan; 3 Kaken Test Center, Tokyo, Japan; 4 Faculty of Textile Science and Technology, Shinshu University, Nagano, Japan; 5 Azearth Corporation, Tokyo, Japan; 6 Department of Ophthalmology, Kitasato University School of Medicine, Kanagawa, Japan; L V Prasad Eye Institute, INDIA

## Abstract

This study aimed to investigate the visibility of colors in congenitally color vision defect people using general and fluorescent colors in an environment simulating sunset to examine the standards for high-visibility safety clothing for general users. Twenty participants with normal trichromats, seven protanopes, and five deuteranopes were included, with mean ages (± standard deviation) of 21.0±1.0, 46,7±16.1, and 56.6±6.9 years, respectively. Dyed fabrics were used to evaluate visibility. We evaluated brightness and conspicuousness sensitivity by combining red, yellow-red, yellow, green, red-purple, blue, white, black, fluorescent yellow, and fluorescent orange. For brightness sensitivity, the combination of fluorescent yellow and white/yellow stripes was highly visible and significantly different from all other samples (p < 0.05). For conspicuousness sensitivity, the combinations of black/fluorescent yellow, black/yellow, black/white, black/yellow-red, and white/red-purple stripes were highly visible and significantly different from all the other samples (p < 0.05). Yellow light is most visible and even better when fluorescent. They are based on specific spectral sensitivity, and yellow is the most visible, even for congenitally colorblind individuals. Furthermore, with regard to color combinations, it was found that the contrast between two distinct light or dark colors, such as black, yellow, black, and white, is perceived to be equally noticeable by congenital color vision defect individuals. This suggests the possible further applications of safety clothing.

## Introduction

High-visibility safety clothing is widely used in construction, road work, airports, and other sites with a high risk of accidents. High visibility safety clothing as “International Organization for Standardization (ISO) 20471 High Visibility Clothing” was established by the ISO in 2013 [1]. However, the target of ISO 20471 is workers in high risk environments, and not workers in other environments or general users in medium or low-risk situations such as pedestrians. Therefore, it is important to create and spread ISO standards for high-visibility safety clothing that can be used for general users to ensure the safety of pedestrians and reduce the risk of accidents. Therefore, we are seeking to develop a standard for high-visibility safety clothing for school-age children with low awareness of risk management, with the aim of reducing traffic accidents [2].

However, only fluorescent colors (fluorescent yellow, fluorescent orange-red, and fluorescent red) are specified in the current ISO standards. Therefore, when developing a color scheme, the number of colors is small, and color diversity is required. In addition, because different colors have different visibilities, it is necessary to investigate which colors will enhance the wearer’s visibility. Studies on field-based colors have reported that drivers are most likely to spot pedestrians wearing fluorescent orange-red vests [3]. However, image analysis suggests that the three fluorescent colors specified, including orange, tend to assimilate with the background color of work sites during the day, resulting in poor visibility [4,5]. Because color plays an important role in attracting visual attention, it is necessary to determine colors that stand out and contrast well rather than relying on bright, easy-to-see colors [6].

These studies have generally not investigated visibility in a congenital colored vision defect. The prevalence of congenital color vision defects is 8% in men and 0.5% in women [7], which is not a small number and needs to be considered.

We found that traffic accidents among school-aged children in Japan occur most frequently between 4 and 6 p.m. [8]. For details on the number of injured school-age children, as sorted by the time of day, please refer to S1 Fig. In this study, we aimed to investigate the visibility of fluorescent and general colors for people with protanopia and deuteranopia in an environment that simulates the luminance, illuminance, and color temperature of sunset between 4 and 6 p.m.

## Methods

### Participants

Twenty healthy participants without ocular diseases, except for refractive errors (seven males and 13 females), seven protanope participants (seven males), and five deuteranope participants (four males and one female) were included. Their mean age (SD) was 21.0±1.0 years, 46,7±16.1 years, and 56.6±6.9 years, respectively. All participants had corrected visual acuity of >20/20. The mean spherical refractive errors for the normal trichromats, protanope, and deuteranope participants were -2.45±2.66 (-0.25 to -6.00) diopter (D) -2.81±2.39 (-0.25 to -5.50) D and -2.45±2.91 (-1.00 to -6.00) D, respectively, and the mean astigmatism results were -0.72±0.63 D, -0.56±0.81 D, and -0.25±0.27 D, respectively. Refractive errors were fully corrected before the study was performed, and participants with astigmatism greater than -2.00 D were excluded. Congenital color vision defect in protanope and deuteranope participants were diagnosed using an anomaloscope. A cone contrast test was performed on the ColorDX CCT-HD (Konan Medical, Inc., CA, US) to account for the effect of the acquired color vision defect due to yellowing of the crystalline lens. Participants were excluded if their S-cone contrast sensitivity score was less than 75, which is diagnostic of color vision defects (details of each participant’s cone contrast score are shown in S2 Fig). Individuals with a history of ophthalmic surgery, retinal or optic nerve disease, or systemic diseases were excluded. This study was conducted in accordance with the principles of the Declaration of Helsinki. The procedures used were approved by the Ethics Committee of the School of Allied Health Sciences of Kitasato University (2019-018B). Written informed consent was obtained from all participants. The study was conducted in accordance with the guidelines and regulations related to informed consent. In addition, we obtained informed consent from the participants to submit the research results to open-access publications, which may lead to personal identification.

### Research samples

**Fig 1:**
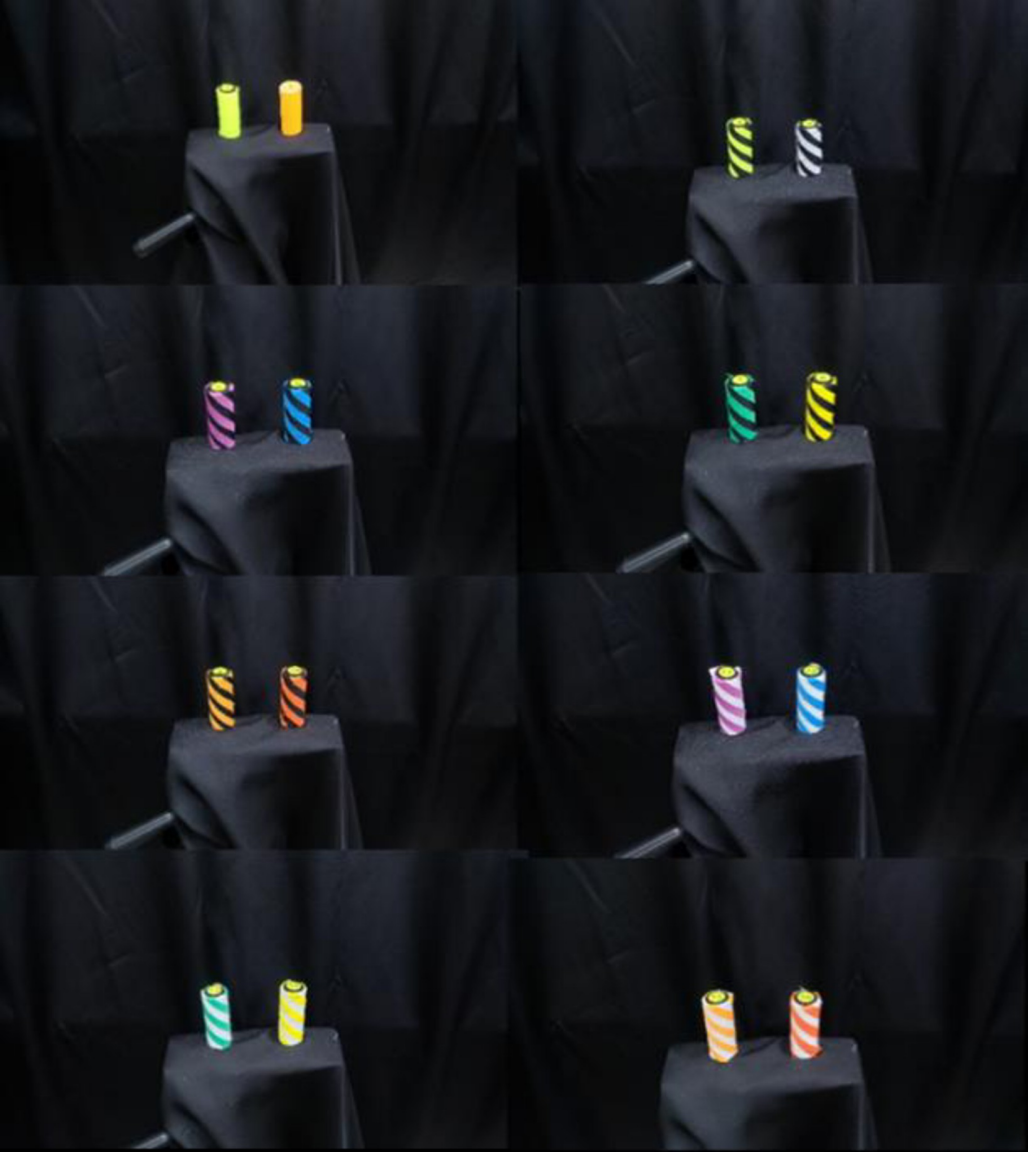
Visibility evaluation samples with a total of 16 different color combinations.

All samples used in this study were made of 100% polyester fabric manufactured by the Sakai Ovex Corporation (Fukui, Japan). A polyvinyl chloride pipe 70 mm in height and 25 mm in diameter was covered by the samples. General and fluorescent colors within the chromaticity coordinate standards for safety colors of ISO 3864–4:2011(en) were selected to undergo visibility evaluation [9]. The colors used were red (x = 0.537, y = 0.366, β = 0.17), yellow (x = 0.445, y = 0.497, β = 0.49), yellow-red (x = 0.500, y = 0.420, β = 0.32), green (x = 0.248, y = 0.423, β = 0.22), blue (x = 0.195, y = 0.219, β = 0. 15), red-purple (x = 0.343, y = 0.247, β = 0.12), white (x = 0.310, y = 0.325, β = 0.64), black (x = 0.314, y = 0.326, β = 0.02), fluorescent yellow (x = 0.375, y = 0.532, β = 1.00), and fluorescent orange (x = 0.553, y = 0.415, β = 0.69). Each color was combined with black and white to form a striped pattern. There were 16 different color combinations: black/florescent yellow stripe, black/white stripe, black/red-purple stripe, black/blue stripe, black/green stripe, black/yellow stripe, black/yellow-red stripe, black/red stripe, white/red-purple stripe, white/blue stripe, white/green stripe, white/yellow stripe, white/yellow-red stripe, white/red stripe, plain fluorescent yellow, and plain fluorescent orange (Fig 1). The stripe pattern was set to 6.0 cycles/degree (c/deg), which is the spatial frequency band of the peak contrast sensitivity, and the ratio of the stripes of the two colors was set to 50%. Sixteen different combinations of samples were compared with 120 patterns. The luminance values of each of the 16 samples were measured using a spot-type single-lens reflex digital luminance meter LS-100 (Konica Minolta, Tokyo, Japan) in a research environment that simulated sunset. Three measurements were taken at a distance of 5.0 m from the sample, each at a measurement angle of 1°, and the average value of each measurement was calculated (Table 1).

**Table 1:** Measured luminance values under sunset environment at the time of measurement.

NO	Color and type of evaluation sample	Average luminance (cd/m^2^)
1	Fluorescent yellow solid color	18.14
2	Fluorescent orange solid color	13.13
3	Black / Fluorescent yellow stripe	8.05
4	Black / White Stripe	7.49
5	Black / Red Purple Stripe	2.57
6	Black/Blue Stripe	2.2
7	Black/Green Stripe	3.2
8	Black/Yellow Stripe	7.31
9	Black / Yellow-Red Stripe	5.47
10	Black/Red Stripe	3.44
11	White / Red Purple Stripe	10.23
12	White / Blue Stripe	9.25
13	White / Green Stripe	11.31
14	White / Yellow Stripe	16.14
15	White / Yellow-Red Stripe	13.62
16	White / Red Stripe	10.63

### Research environment

**Fig 2:**
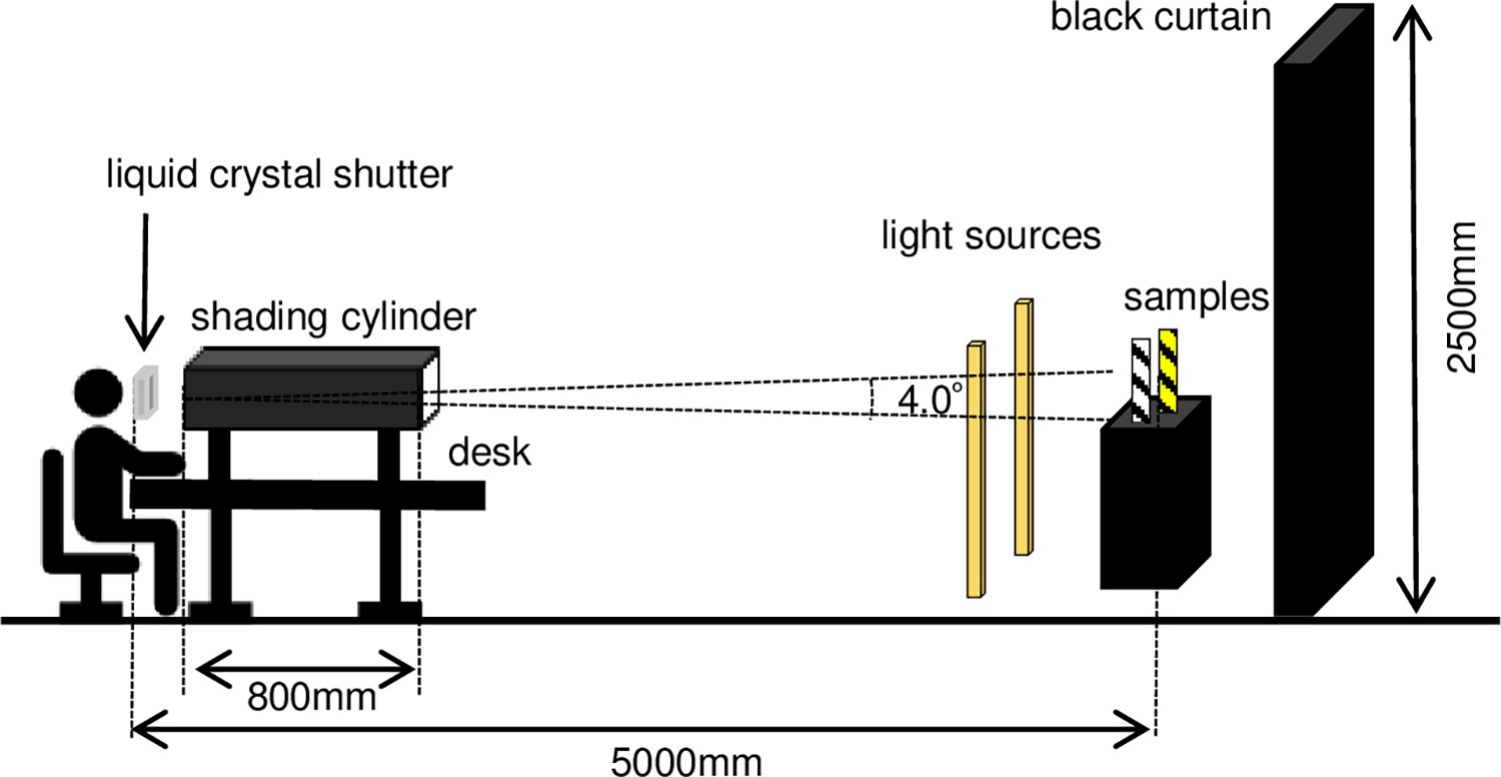
Research environment. Two evaluation samples were placed 5 m in front of the participant in a dark room.

**Fig 3:**
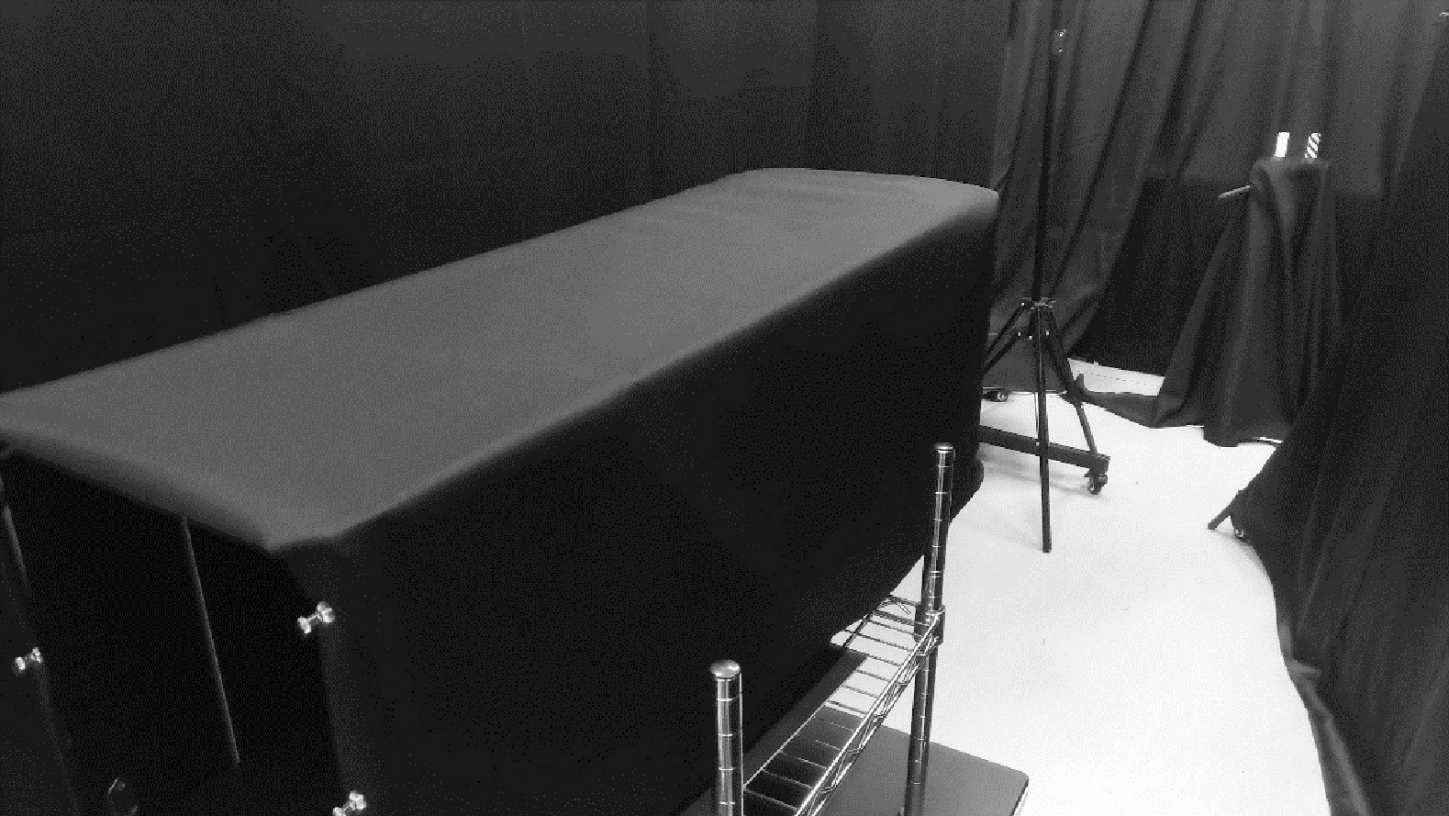
Experimental scene. The sunset environment was recreated by covering the participants’ surroundings with a blackout curtain. (This photo was taken in a bright room).

The participants were surrounded by a black curtain (approximately 2.5 m) to prevent uneven luminance distribution in the visual field. To reproduce the brightness at sunset in a dark room, a light source was placed in front of the sample so that it was not visible to the examinee, and the visual target illuminance was set to 300 lx (Figs 2 and 3). SFX-502 (Panasonic, Osaka, Japan), a warm white light-emitting diode with a color temperature of approximately 2700 K, was used as the light source. To fix the spatial frequency of the samples, two different samples were placed 5 m away from the line of sight, and two light sources were placed in front of the samples. These simulations of the lighting environment at sunset are based on measured luminance and illuminance color temperatures during actual sunset hours, averaged over weather and interday variations.

### Measurement method

**Fig 4:**
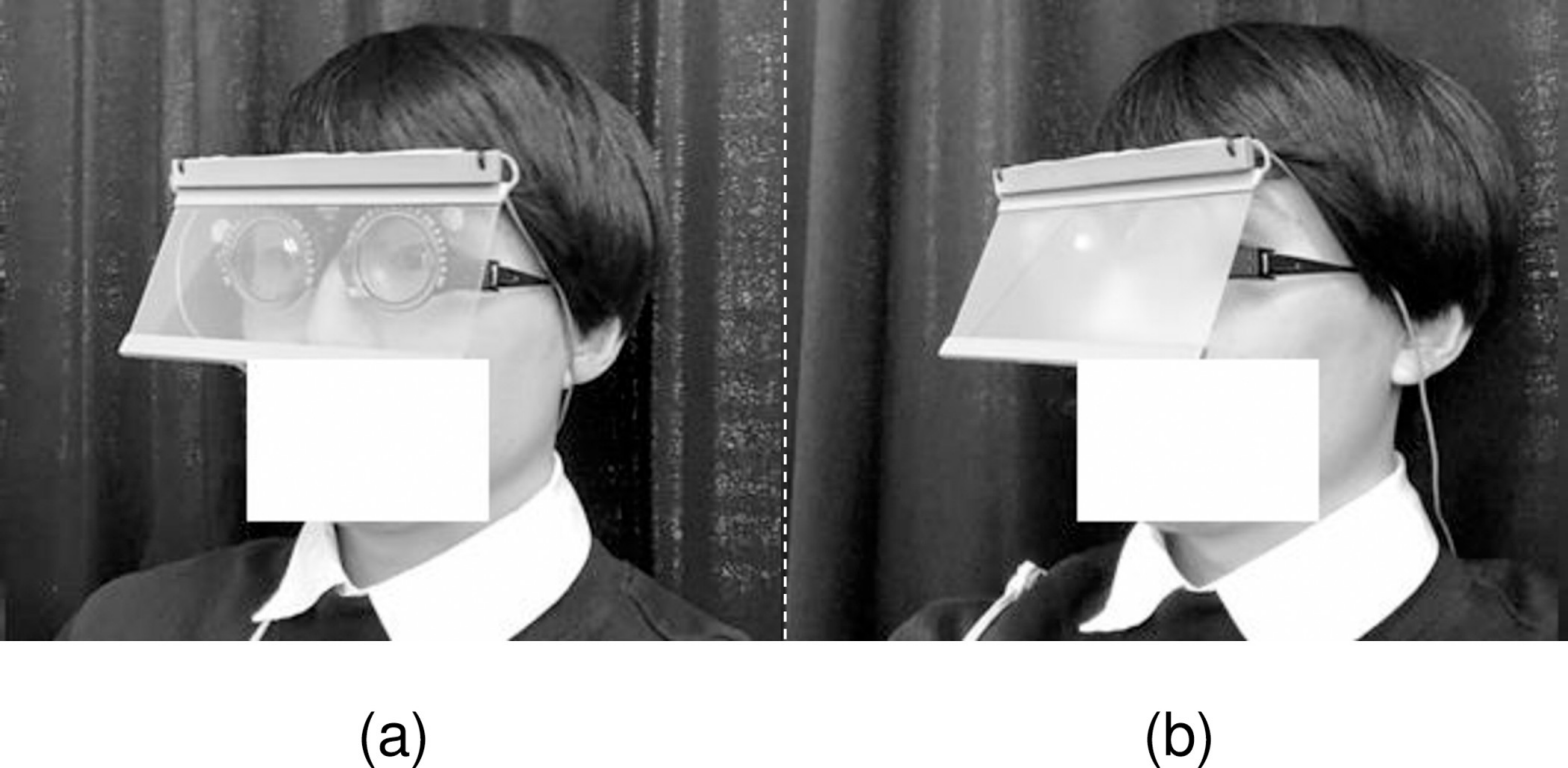
Liquid crystal shutter. Electronically controlled to open (a) and close (b).

First, the refractive error was fully corrected in a light room, and basic data such as the presence or absence of the participant’s medical history, were obtained. Next, we evaluated the visibility of the samples in a simulated sunset environment. For the visibility evaluation, participants were seated, a liquid crystal shutter (Koyo Corporation, Singapore) was placed in front of their eyes, and a shading cylinder with a 4° field of view was placed behind it (Fig 2). By applying this liquid-crystal shutter voltage, the shutter can be opened and closed to instantly change the view from invisible to visible. When the shutter was open, the parallel transmittance was 74%, and when it was closed, the parallel transmittance was 5% (Fig 4). The liquid crystal shutter was connected to a PC and software with a control signal generator AWG10K (Elmos Co., Ltd., Aichi, Japan) and a semiconductor relay AC100SSR (Asakusa Giken Co., Ltd., Tokyo, Japan). The signals were controlled using the dedicated Waveform Editing software (version 1.1, Elmos Co., Ltd.). The open/close function of the shutter was used to show the samples for 0.5 s. This time was based on “the time it takes for a car traveling at 50 km/h to recognize a person 50 m ahead, consider the braking distance of the vehicle, and stop approximately 10 m in front of the person [10]”.

**Fig 5:**
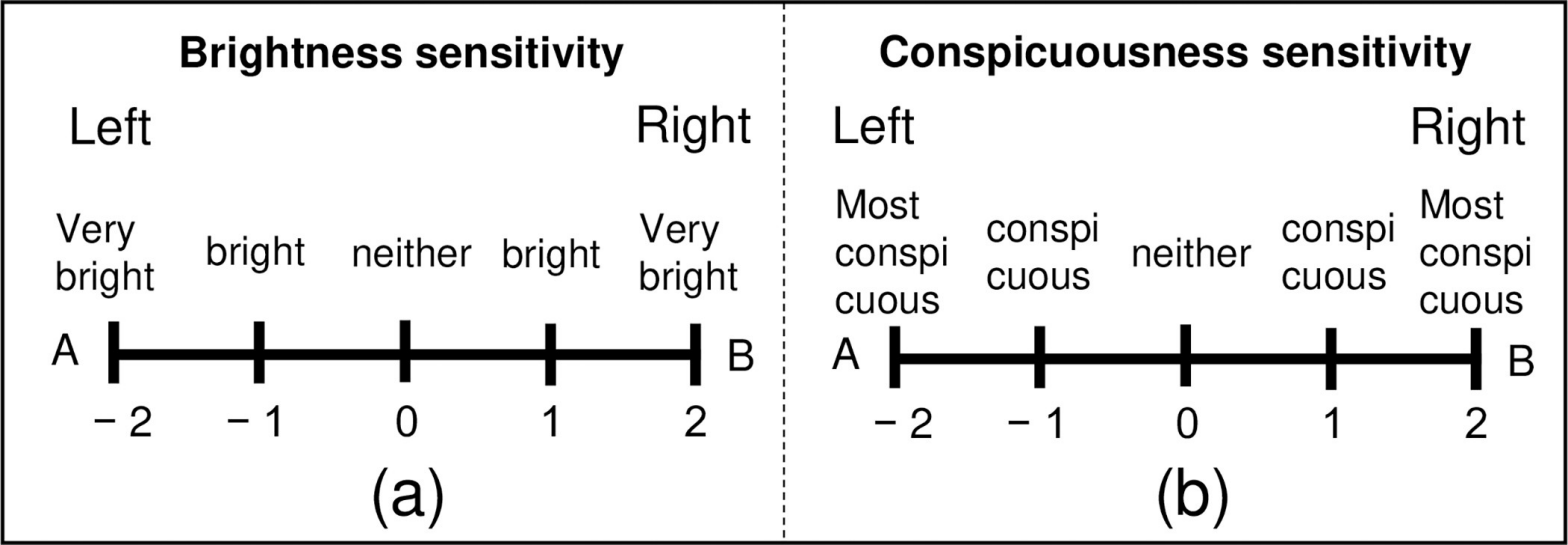
Evaluation items (English version). (a) Brightness sensitivity evaluation sheet. (b) Conspicuousness sensitivity evaluation sheet. The two samples were compared, with the item circled having the highest brightness and conspicuity.

Two combinations of the left and right samples were randomly presented 5 m in front of the participant to evaluate visibility. The left and right samples were evaluated by circling which sample was brighter or more conspicuous as A and B, as shown in Fig 5. Only brightness sensitivity was evaluated in participants with normal trichromats, and in participants with protanopes and deuteranopes, brightness and conspicuity sensitivity were evaluated. Brightness and conspicuousness sensitivity measurements were evaluated separately in the evaluation experiments. Comparing the two samples as A and B, A’s (or B’s) degree of brightness was answered with “very bright” or “bright.” When the left and right samples appeared to be equally bright, or when it was impossible to judge which was brighter, the participants were asked to select “neither.” The conspicuousness of the samples was also evaluated in the same way. The time available to view the two samples was approximately 0.5 s when the liquid crystal shutter was open. All participants were given breaks to ensure sufficient time to fill out the evaluation sheet and avoid fatigue.

### Statistical analysis

Statistical analysis was conducted using Excel 2016 (Microsoft Co., Albuquerque, NM, USA) and Bell Curve for Excel (Social Research Information, Inc., Tokyo, Japan). The analysis was performed using Sheffé’s (Nakaya) paired comparison method. This is a grading method in which any two objects are taken and compared on a one-to-one basis, and the results of all comparisons are evaluated collectively. Analysis of variance (ANOVA) and the average degree of preference corresponding to the rated value of each sample were obtained. Based on the results of the analysis of variance, it was determined that there is a significant difference between samples when the p-value of the main effect is less than 0.05. In this case, a significant difference existed if the difference in the average degree of preference between samples was greater than the yardstick value (Yφ). The yardstick value (Yφ) was obtained using Eq (1).


$$Y_{\varphi }=q_{\varphi }(t,f_{e})\sqrt{\frac{V_{e}}{tn}}.$$ (1)

φ is the significance level to be tested and 0.05 is used. t and n are the numbers of samples and participants to be evaluated, respectively. f_e_ and V_e_ are the degrees of freedom and variance of the residuals, respectively, obtained from the analysis of the variance table. q_φ_(t, f_e_) was also studentized at a significance level of 0.05. For all tests, a P value < 0.05 was considered statistically significant.

## Results

### Brightness sensitivity

**Fig 6:**
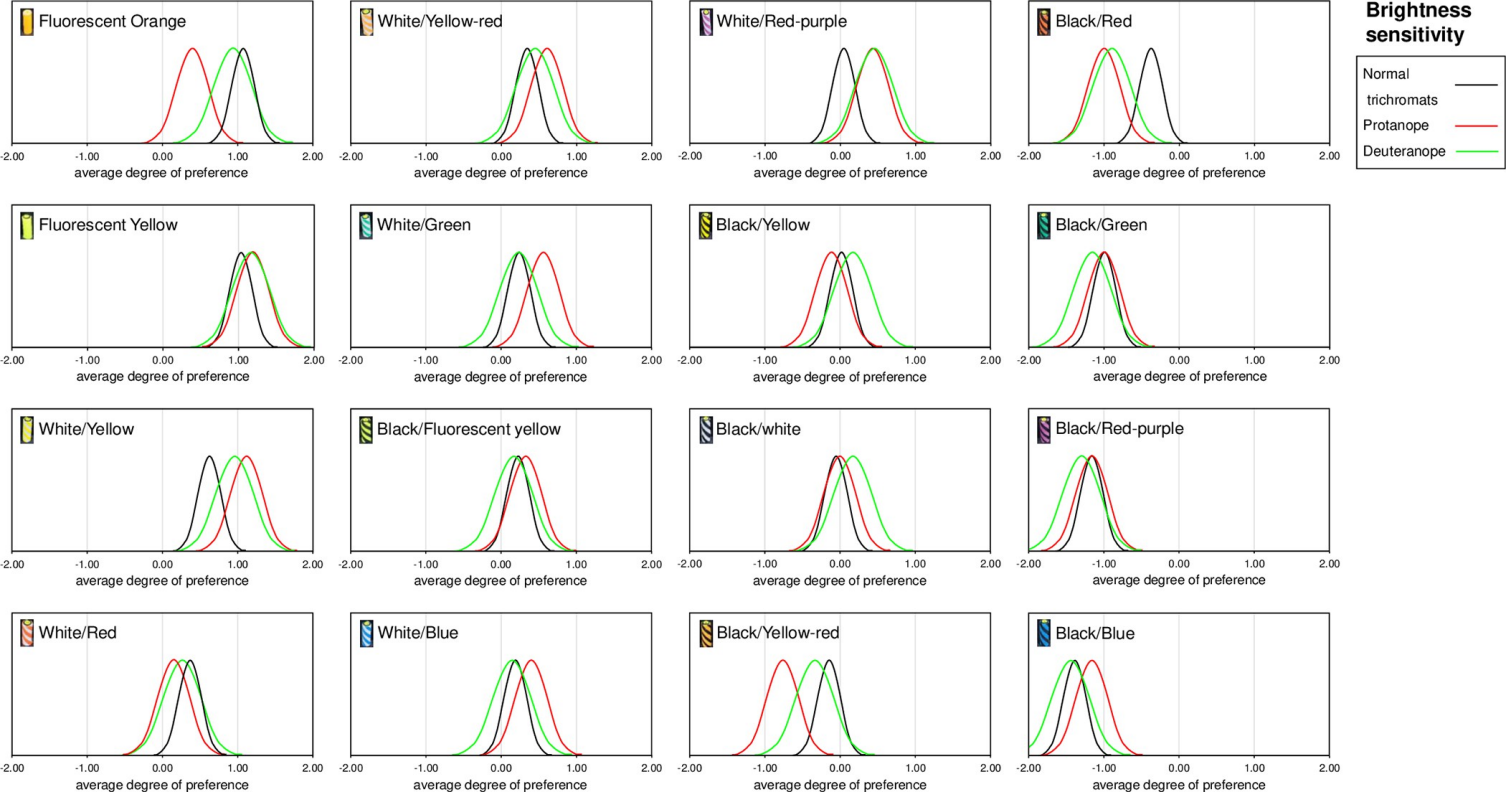
Bell curve of average degree of preference (brightness sensitivity) for normal trichromats and protanope and deuteranope. The average degree of preference for brightness sensation for normal trichromats, protanopes, and deuteranopes is shown in a standard normal distribution for each sample. For fluorescent yellow, the three groups have a high average degree of preference and the bell curves almost overlap. However, in fluorescent orange, the average degree of preference for protanope is lower than the others, and the bell curve is shifted in the negative direction.

Table 2 shows the analysis of variance table for normal trichromats, and protanope and deuteranope using the Scheffé method. The F-test results showed significant differences in the main effects for all groups. Since significant differences were found between normal trichromats and protanope and deuteranope, the yardstick values (Y) obtained using q-values from the studentized range with a 5% level of significance for the degrees of freedom of each error, and the average degree of preference and confidence interval for each sample are listed in Table 3. Normal trichromats had the highest average degree of preference for fluorescent orange at 1.08, with a difference of 0.05 from fluorescent yellow, which is less than the yardstick value of 0.32, and is therefore equally visible. All samples with a lower average degree of preference than fluorescent yellow were significantly different (p < 0.05) from the average degree of preference for fluorescent orange, as they differed by more than 0.32. Protanope had the highest average degree of preference for fluorescent yellow at 1.20, with a difference of 0.09 from the white/yellow, which is less than the yardstick value of 0.47 and therefore of equal visibility. All samples with a lower average degree of preference than the white/yellow were significantly different (p < 0.05) from the fluorescent yellow average degree of preference, as they differed by more than 0.47. The deuteranope had the highest average degree of preference for fluorescent yellow at 1.17, with a difference of 0.22 from white/yellow, which is less than the yardstick value of 0.48 and therefore of equal visibility. All samples with a lower average degree of preference than white/yellow were significantly different (p < 0.05) from the fluorescent yellow average degree of preference, as they differed by more than 0.48. Fig 6 presents a comparison of sample results for all three of these groups with a standardized normal distribution. The distribution is relatively similar for normal trichromats and deuteranope, but the distribution is off for the protanope, especially the low average degree of preference for fluorescent orange.

**Table 2:** Analysis-of-variance tables for Brightness sensitivity in the normal trichromats, protanope, and deuteranope using Scheffé’s method.

	Factorial variation	Sum of squares (S)	Degrees of freedom (fd)	Variance(V)	Ratio of variance (F)	P value
**Normal trichromats**	**Total (T)**	5560	2400			
	**Main effect**	2384	15	158.94	122.13	< 0.001***
	**Main effect×personal**	2157	60	35.95	27.62	< 0.001***
	**Combined effect**	209	105	1.99	1.53	< 0.001***
	**Error €**	2967	2280	1.30		
**Protanope**	**Total (T)**	1915	840			
	**Main effect**	1036	15	69.09	64.60	< 0.001***
	**Main effect×personal**	1137	60	18.95	17.72	< 0.001***
	**Combined effect**	109	105	1.04	0.97	0.57
	**Error €**	770	720	1.07		
**Deuteranope**	**Total (T)**	1145	600			
	**Main effect**	687	15	45.78	58.31	< 0.001***
	**Main effect×personal**	832	60	13.87	17.66	< 0.001***
	**Combined effect**	82	105	0.78	0.99	0.51
	**Error €**	377	480	0.79		

Significant differences (***) were found for the main effect in all groups using the F-test (p < 0.001).

**Table 3:** Results of 95% confidence intervals and average degree of preference (Brightness sensitivity) for the sample using Scheffé’s method.

Y_φ_	sample	average degree of preference (Brightness sensitivity)	Confidence interval	
			upper (95%)	lower (95%)
**Normal trichromats**	Fluorescent Orange	1.08	1.40	0.77
(Y_0.05_ = 0.32)	Fluorescent Yellow	1.03	1.35	0.72
	White/Yellow	0.62	0.93	0.30
	White/Red	0.36	0.68	0.05
	White/Yellow-red	0.33	0.65	0.02
	White/Green	0.24	0.55	-0.08
	Black/Fluorescent yellow	0.23	0.55	-0.08
	White/Blue	0.18	0.50	-0.13
	White/Red-purple	0.03	0.35	-0.28
	Black/Yellow	0.02	0.33	-0.30
	Black/white	-0.05	0.26	-0.37
	Black/Yellow-red	-0.15	0.17	-0.47
	Black/Red	-0.37	-0.06	-0.69
	Black/Green	-0.99	-0.68	-1.31
	Black/Red-purple	-1.17	-0.86	-1.49
	Black/Blue	-1.39	-1.07	-1.70
Protanope	Fluorescent Yellow	1.20	1.68	0.71
(Y_0.05_ = 0.47)	White/Yellow	1.11	1.59	0.62
	White/Yellow-red	0.61	1.09	0.12
	White/Green	0.56	1.05	0.08
	White/Red-purple	0.43	0.91	-0.06
	Fluorescent Orange	0.40	0.89	-0.08
	White/Blue	0.40	0.89	-0.08
	Black/Fluorescent yellow	0.32	0.81	-0.16
	White/Red	0.16	0.64	-0.32
	Black/white	0.00	0.48	-0.48
	Black/Yellow	-0.11	0.38	-0.59
	Black/Yellow-red	-0.77	-0.28	-1.25
	Black/Green	-1.00	-0.52	-1.48
	Black/Red	-1.00	-0.52	-1.48
	Black/Blue	-1.15	-0.67	-1.64
	Black/Red-purple	-1.16	-0.68	-1.64
Deuteranope	Fluorescent Yellow	1.17	1.77	0.57
(Y_0.05_ = 0.48)	White/Yellow	0.95	1.55	0.35
	Fluorescent Orange	0.94	1.54	0.34
	White/Red-purple	0.45	1.05	-0.15
	White/Yellow-red	0.44	1.04	-0.16
	White/Red	0.27	0.87	-0.34
	White/Green	0.25	0.85	-0.35
	Black/Fluorescent yellow	0.17	0.77	-0.43
	Black/white	0.17	0.77	-0.43
	Black/Yellow	0.17	0.77	-0.43
	White/Blue	0.14	0.74	-0.46
	Black/Yellow-red	-0.34	0.26	-0.94
	Black/Red	-0.89	-0.29	-1.49
	Black/Green	-1.16	-0.56	-1.76
	Black/Red-purple	-1.30	-0.70	-1.90
	Black/Blue	-1.44	-0.84	-2.04

There is a significant difference (p < 0.05) when the difference in average degree of preference between samples is greater than the Yardstick value (Yφ).

### Conspicuousness sensitivity

**Fig 7:**
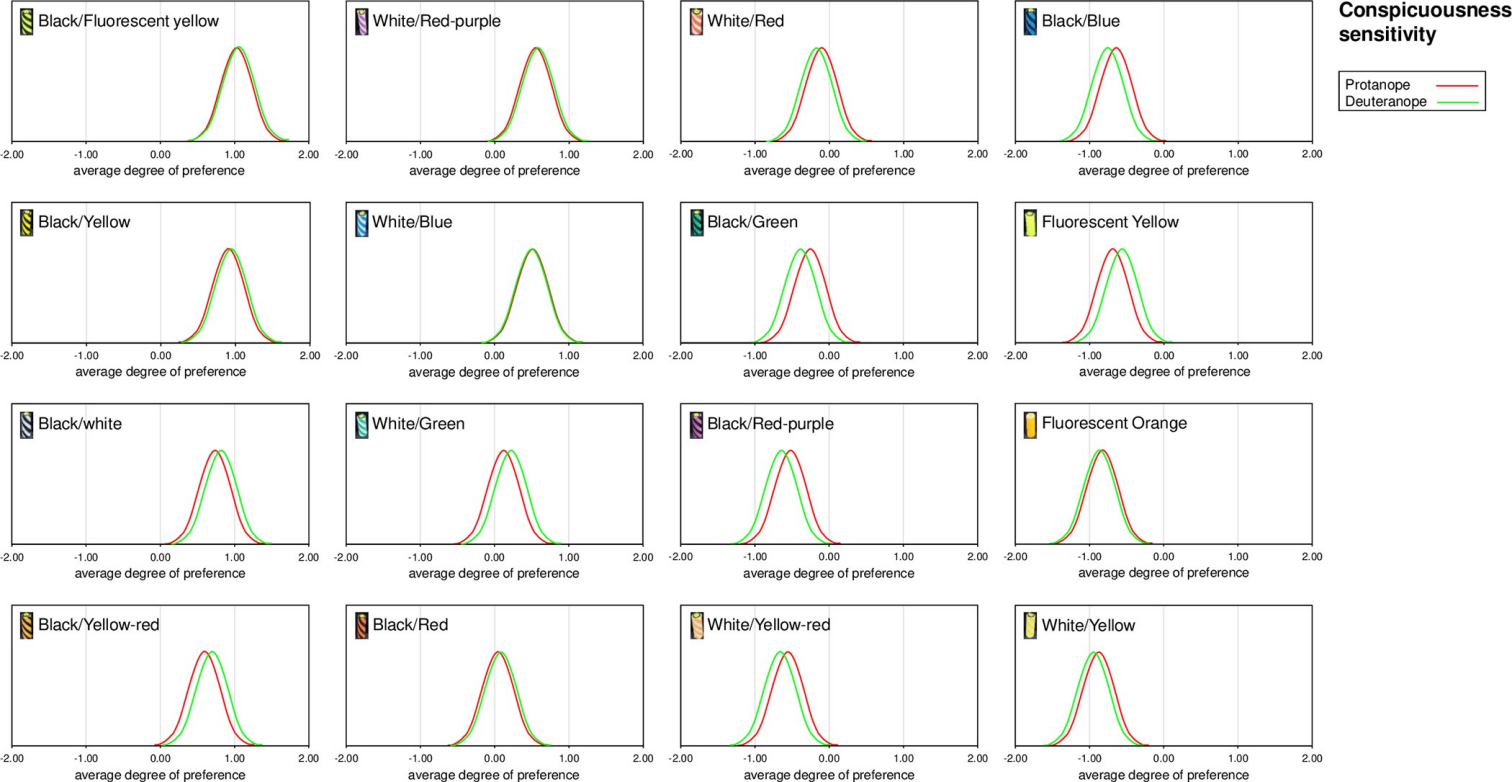
Bell curve of average degree of preference (conspicuousness sensitivity) for protanope and deuteranope. The average degree of preference for the sense of conspicuousness for protanope and deuteranope is shown in a standard normal distribution for each sample. The bell curves overlap in almost all samples in the same way, with comparable visibility.

**Fig 8:**
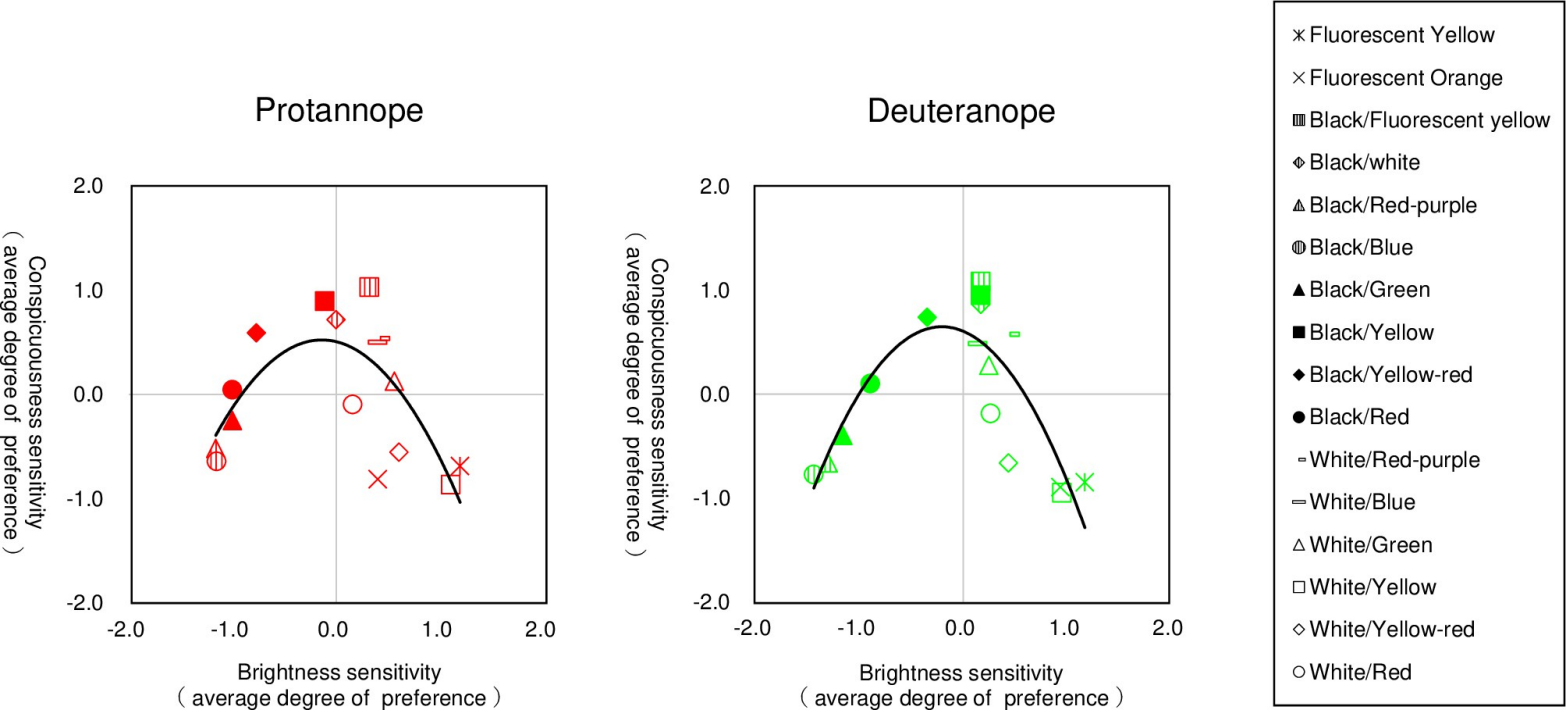
Scatterplot of brightness sensitivity and conspicuousness sensitivity. The vertical axis represents the average degree of preference for the sense of conspicuousness, and the horizontal axis represents the average degree of preference for the sense of brightness. Black/fluorescent yellow is highly visible because it is bright and easily noticeable, while black/blue is less visible because it is dark and less noticeable. There is no correlation between brightness sense and conspicuousness sense.

Table 4 shows the analysis of variance table for protanopes and deuteranopes using the Scheffé method. The F-test results showed significant differences in the main effects for all groups. Since significant differences were found between the rotanope and deuteranope, the yardstick values (Y) were obtained using q-values from the Studentized range with a 5% level of significance for the degrees of freedom of each error; the average degree of preference and confidence interval for each sample are presented in Table 5. Protanope had the highest average degree of preference for black/fluorescent yellow at 1.03, with a difference of 0.14 from the black/yellow, 0.32 from the black/white, 0.44 from the black/yellow-red, and 0.49 from white/red-purple, which is less than the yardstick value of 0.51 and therefore equally visible. All samples with a lower average degree of preference than white/red-purple were significantly different (p < 0.05) from the black/fluorescent yellow average degree of preference, as they differed by more than 0.51. Deuteranope had the highest average degree of preference for black/fluorescent yellow at 1.08, with a difference of 0.13 from black/yellow, 0.22 from the black/white, 0.34 from the black/yellow-red, 0.50 from the white/red-purple, which is less than the yardstick value of 0.50, and therefore equally visible. All samples with a lower average degree of preference than white/red-purple were significantly different (p < 0.05) from the black/fluorescent yellow average degree of preference, as they differed by more than 0.50. Fig 7 compares the sample results for all three groups with a standardized normal distribution. The protanopes and deuteranopes showed similar normal distributions in almost all samples. We analyzed the correlation between brightness and conspicuousness sensitivity, but there was no correlation with either the protanope or deuteranope (Fig 8).

**Table 4:** Analysis-of-variance tables for Conspicuousness sensitivity in the protanope and deuteranope using Scheffé’s method.

	Factorial variation	Sum of squares (S)	Degrees of freedom (fd)	Variance(V)	Ratio of variance (F)	P value
**Protanope**	**Total (T)**	1661	840			
	**Main effect**	695	15	46.34	38.57	< 0.001***
	**Main effect×personal**	563	60	9.38	7.81	< 0.001***
	**Combined effect**	101	105	0.96	0.80	0.93
	**Error €**	865	720	1.20		
**Deuteranope**	**Total (T)**	1099	600			
	**Main effect**	614	15	40.91	49.99	< 0.001***
	**Main effect×personal**	586	60	9.76	11.93	< 0.001***
	**Combined effect**	93	105	0.88	1.08	0.30
	**Error €**	393	480	0.82		

Significant differences (***) were found for the main effect in all groups using the F-test (p < 0.001).

**Table 5:** Results of 95% confidence intervals and average degree of preference(Conspicuousness sensitivity) for the sample using Scheffé’s method.

Y_φ_	sample	average degree of preference (Conspicuousness sensitivity)	Confidence interval	
			upper (95%)	lower (95%)
Protanope	Black/Fluorescent yellow	1.03	1.54	0.51
(Y_0.05_ = 0.51)	Black/Yellow	0.89	1.41	0.38
	Black/white	0.71	1.23	0.20
	Black/Yellow-red	0.59	1.10	0.08
	White/Red-purple	0.54	1.05	0.02
	White/Blue	0.50	1.01	-0.01
	White/Green	0.13	0.64	-0.39
	Black/Red	0.04	0.56	-0.47
	White/Red	-0.10	0.41	-0.61
	Black/Green	-0.25	0.26	-0.76
	Black/Red-purple	-0.52	-0.01	-1.03
	White/Yellow-red	-0.55	-0.04	-1.07
	Black/Blue	-0.64	-0.13	-1.16
	Fluorescent Yellow	-0.69	-0.17	-1.20
	Fluorescent Orange	-0.81	-0.30	-1.33
	White/Yellow	-0.87	-0.35	-1.38
Deuteranope	Black/Fluorescent yellow	1.08	1.59	0.59
(Y_0.05_ = 0.50)	Black/Yellow	0.95	1.45	0.45
	Black/white	0.86	1.36	0.36
	Black/Yellow-red	0.74	1.24	0.24
	White/Red-purple	0.58	1.08	0.07
	White/Blue	0.49	0.99	-0.01
	White/Green	0.28	0.78	-0.23
	Black/Red	0.10	0.60	-0.40
	White/Red	-0.19	0.31	-0.69
	Black/Green	-0.40	0.10	-0.90
	Fluorescent Yellow	-0.59	-0.09	-1.09
	Black/Red-purple	-0.66	-0.16	-1.16
	White/Yellow-red	-0.66	-0.16	-1.16
	Black/Blue	-0.78	-0.27	-1.28
	Fluorescent Orange	-0.85	-0.35	-1.35
	White/Yellow	-0.95	-0.45	-1.45

There is a significant difference (p < 0.05) when the difference in average degree of preference between samples is greater than the Yardstick value (Yφ).

## Discussion

The purpose of this study was to investigate color visibility in severely congenitally colored vision defects in a sunset-simulated environment. Because we are considering safety clothing that can be recommended for school-age children.

### Brightness sensitivity

The visibility of normal trichromats to fluorescent colors was consistent with the reported field-based visibility, with orange being the most visible fluorescent color. However, no difference was observed between fluorescent colors, but was comparable to fluorescent yellow [4,11]. These color visibilities are roughly in line with the specific spectral sensitivity reported by the Commission Internationale de l’Eclairage, rather than the luminance of the cloth [12]. Fluorescent orange, which is the most visible color to people with normal trichromats, showed similar results in deuteranopes. However, fluorescent yellow was similar in protanopes, but fluorescent orange was less visible, with the same average degree of preference as the less brightly perceived white/short-wavelength and black combined samples. Protanopes lack long wave-sensitive cones [13], which suggests that long-wavelength colors such as red and orange are perceived as darker, and not only fluorescent orange but also the visibility of the combination of white and red had as lower average degree of preference than normal trichromats. Therefore, protanope visibility should be a primary consideration when producing color designs; yellow is most suitable, and fluorescent yellow is even better.

### Conspicuousness sensitivity

The visibility of conspicuousness sensitivity in color vision defect is very similar to the results we previously reported for normal trichromats [2]. In people with a color vision defect, the larger the contrast difference in brightness between the two-color combinations, the higher the visibility result.

Considering both protanopes and deuteranopes, black/fluorescent yellow was found to be the most visible, followed by black/yellow, black/white, black/yellow-red, and white/red-purple; all were equally visible. All of them combine high-and low-brightness colors. Short-wavelength colors were found to be incompatible with black. Combining the two colors with a contrast difference proved to be the best way to improve visibility.

### Potential application in safety clothing

Our study was conducted in a sunset environment. Therefore, the recommended clothing colors are not intended to be effective at night when dark. Rather, our aim is for optimizing the clothing design of school-age children for the situations of traveling to and from school in situations where there is natural light, such as at sunset. In this study, we found that black and fluorescent yellow had the highest visibility, with a contrasting difference between the two colors. It is suggested that wearing clothing of these colors during the day and at sunset improves visibility. Retroreflective materials are not the most visible during the day because they are less luminous than fluorescent yellow or yellow fabrics. At night, retroreflective materials are maximized by automobile headlights; therefore, the use of conventional high-visibility safety clothing is recommended. It is anticipated that the safest approach would be to use clothing based on the results of this study during the day and at sunset, and wearing conventional clothing at night.

A person wearing this combination of clothing during the day or at sunset is easily recognized as an object; however, to be recognized as a person, indirect aspects such as "biological movement" need to be emphasized, and this should be considered when creating high-visibility safety clothing for testing [14].

A limitation of this study is that most of the participants in the present study were middle-aged in their 40s–50s with congenital color vision defects, and the cone contrast test showed a slightly reduced S-cone sensitivity compared to younger participants (S2 Fig). However, since none of the participants had serious acquired or congenital color vision defects in the sensitivity of S-cones, and as per previous reports, the total transmission of light was minimally affected in the 40–59 age group [15]. Further, we believe that the effect of age was small in the present study.

## Supporting information

S1 Fig Number of injuries among school-aged children in Japan by time of day in 2019. Number of school age children in traffic accidents in 2019 by Japan’s National Police Agency. Traffic accidents are most common between 4:00 p.m. and 6:00 p.m.
(PDF)

S2 Fig Results of cone contrast test for normal trichromats and protanope and deuteranope. *: 0.01 < p < 0.05; ***: p < 0.001. Contrast sensitivity (logCS) were converted to scores, and a score of ≥75 was diagnosed as normal trichromats. Long wave-sensitive cone scores were significantly different among all groups (p < 0.001 by Scheffe’s multiple comparisons). The scores were significantly lower in the protanope group, with scores <75. Middle-wave-sensitive cone scores were significantly different among all groups (p < 0.001 by Scheffe’s multiple comparisons). and was significantly lower in deuteranope, with scores <75. Short-wave-sensitive cone(S-cone) scores were significantly different between normal trichromats and protanope (p = 0.011 by Scheffe’s multiple comparisons). However, the protanope scores were >75, and the S-cone sensitivity was normal.
(PDF)
