# Supplementary figures and images for: High visibility colored fabrics for normal trichromats and individuals with color vision defects in a sunset-simulated environment
Source: PLoS One. 2022 Sep 16;17(9):e0274824. doi: 10.1371/journal.pone.0274824 (PMC9480988; doi:10.1371/journal.pone.0274824)

S1 Fig.

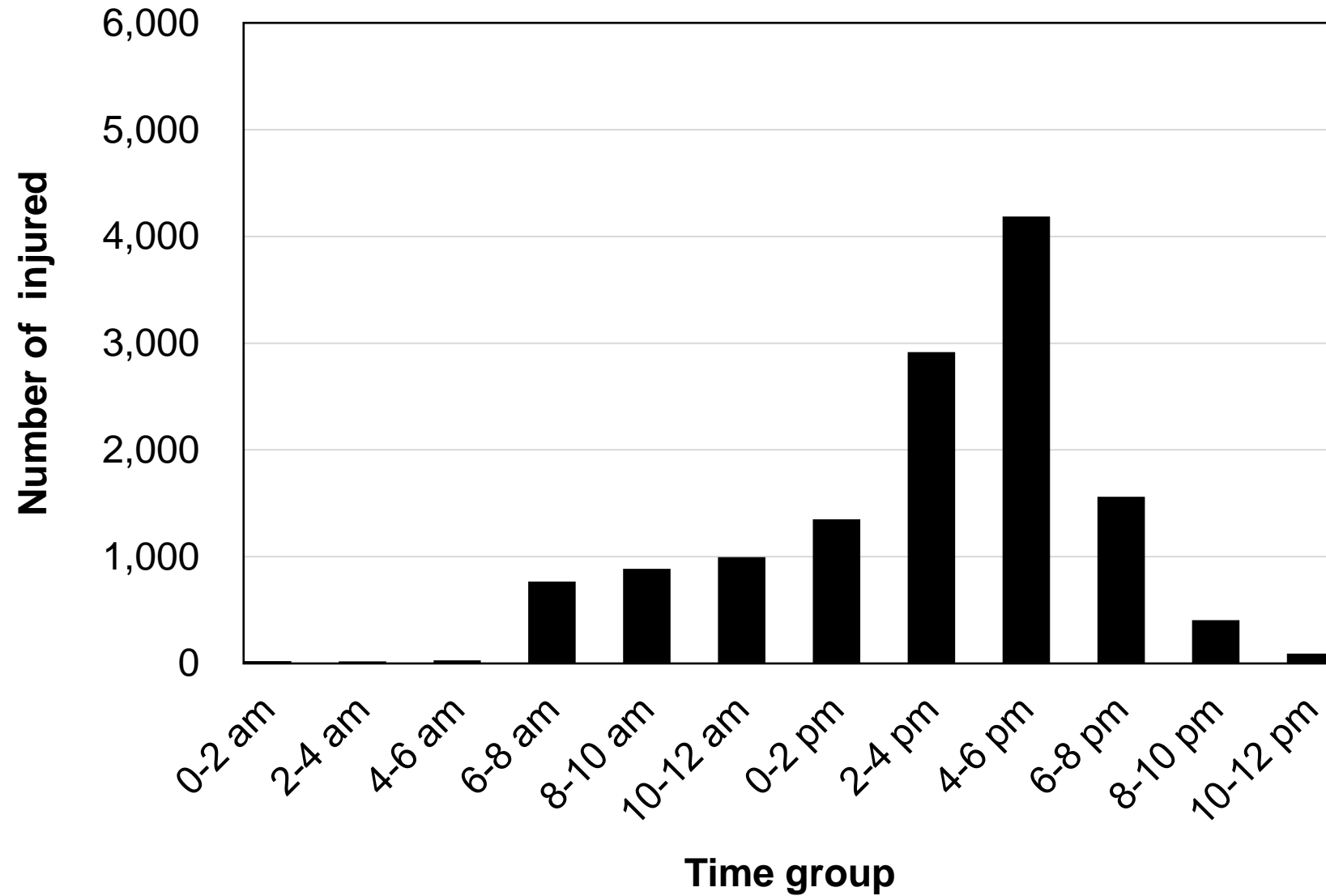

Supplement: S1 Fig — Number of school age children in traffic accidents in 2019 by Japan’s National Police Agency. Traffic accidents are most common between 4:00 p.m. and 6:00 p.m. (PDF) [file pone.0274824.s001.pdf]

# S2 Fig.

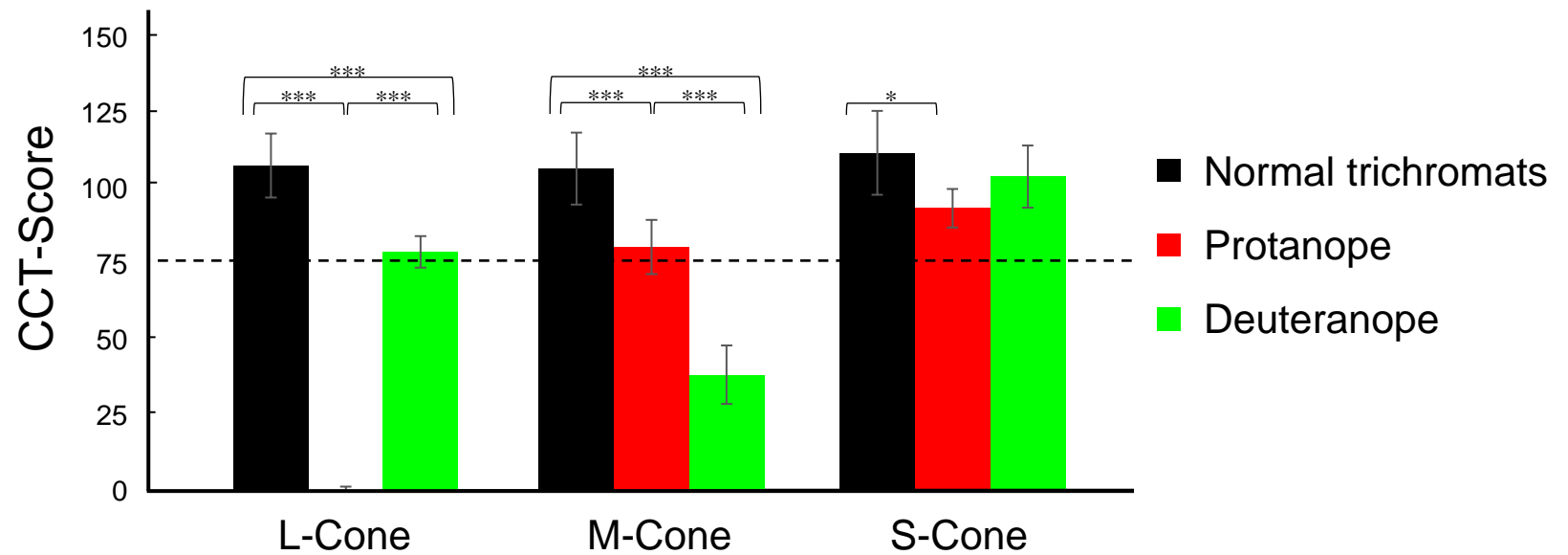

Supplement: S2 Fig — *: 0.01 < p < 0.05; ***: p < 0.001. Contrast sensitivity (logCS) were converted to scores, and a score of ≥75 was diagnosed as normal trichromats. Long wave-sensitive cone scores were significantly different among all groups (p < 0.001 by Scheffe’s multiple comparisons). The scores were significantly lower in the protanope group, with scores <75. Middle-wave-sensitive cone scores were significantly different among all groups (p < 0.001 by Scheffe’s multiple comparisons). and was significantly lower in deuteranope, with scores <75. Short-wave-sensitive cone(S-cone) scores were significantly different between normal trichromats and protanope (p = 0.011 by Scheffe’s multiple comparisons). However, the protanope scores were >75, and the S-cone sensitivity was normal. (PDF) [file pone.0274824.s002.pdf]
